# Peripheral neuropathy in patients with CPEO associated with single and multiple mtDNA deletions

**DOI:** 10.1212/NXG.0000000000000113

**Published:** 2016-10-19

**Authors:** Diana Lehmann, Malte E. Kornhuber, Carolina Clajus, Charlotte L. Alston, Andreas Wienke, Marcus Deschauer, Robert W. Taylor, Stephan Zierz

**Affiliations:** Form the Department of Neurology (D.L., M.E.K., C.C., S.Z.), Institute of Medical Epidemiology, Biometrics and Informatics (A.W.), University of Halle-Wittenberg, Halle/Saale, Germany; Wellcome Trust Centre for Mitochondrial Research (C.L.A., R.W.T.), Institute of Neuroscience, The Medical School, Newcastle University, UK; and Department of Neurology (M.D.), Technical University Munich, Germany.

## Abstract

**Objective:**

To characterize peripheral nerve involvement in patients with chronic progressive external ophthalmoplegia (CPEO) with single and multiple mitochondrial DNA (mtDNA) deletions, based on clinical scores and detailed nerve conduction studies.

**Methods:**

Peripheral nerve involvement was prospectively investigated in 33 participants with CPEO (single deletions n = 18 and multiple deletions n = 15). Clinically, a modified Total Neuropathy Score (mTNS) and a modified International Cooperative Ataxia Rating Scale (mICARS) were used. Nerve conduction studies included Nn. suralis, superficialis radialis, tibialis, and peroneus mot. Early somatosensory evoked potentials were obtained by N. tibialis stimulation.

**Results:**

Participants with multiple deletions had higher mTNS and mICARS scores than those with single deletions. Electrophysiologically in both sensory nerves (N. suralis and N. radialis superficialis), compound action potential (CAP) amplitudes and nerve conduction velocities were lower and mostly abnormal in multiple deletions than those in single deletions. Early somatosensory evoked potentials of N. tibialis revealed increased P40 latencies and decreased N35-P40 amplitudes in multiple deletions. Both sensory nerves had higher areas under the receiver operating characteristic curves for the decreased CAP amplitudes than the 2 motor nerves. The N. suralis had the best Youden index, indicating a sensitivity of 93.3% and a specificity of 72.2% to detect multiple deletions.

**Conclusions:**

Peripheral nerve involvement in participants with multiple mtDNA deletions is an axonal type of predominant sensory neuropathy. This is clinically consistent with higher mTNS and mICARS scores. Sensory nerve involvement in participants with multiple deletions was not correlated with age at onset and duration of disease.

Chronic progressive external ophthalmoplegia (CPEO) is the main clinical symptom associated with single, large scale, and multiple deletions of mitochondrial DNA (mtDNA). Peripheral neuropathy is a typical but variably expressed additional symptom.^1–3^ However, reports about peripheral neuropathy in patients with single deletions are rare.^4–6^ Electrophysiologic studies in mitochondrial diseases usually revealed a predominantly axonal type of nerve fiber damage.^7–15^ However, a predominantly demyelinating pattern or a mixed axonal and demyelinating type of peripheral nerve involvement has also been found.^4,11,16^ Sural nerve biopsies revealed axonal degeneration^12,13^ or demyelination.^11^ It has already been shown that patients with multiple deletions (clinically SANDO [sensory ataxic neuropathy with dysarthria and ophthalmoparesis] syndrome) had reduced compound action potential (CAP) amplitudes in predominantly sensory nerves. Clinically, they might often present as SANO or SANDO syndrome.^17^ There is only one previous study systematically comparing peripheral nerve involvement in patients with CPEO with single and multiple mtDNA deletions.^18^ In that study, it has been postulated that peripheral neuropathy is a rare finding in patients with CPEO with single deletions compared with that in patients with CPEO with mtDNA point mutations, multiple mtDNA deletions, and nuclear defects. However, in that study, only one patient with single mtDNA deletion and peripheral nerve involvement has been investigated. Four other patients had the typical mitochondrial encephalomyopathy, lactic acidosis, and stroke-like episode mtDNA point mutation. Nine other patients had the nuclear POLG mutation, but multiple deletions have not been analyzed in 3/9 patients. In 2 patients with multiple deletions, the nuclear defect has not been identified. In all these patients, no detailed clinical and neurophysiologic data were presented, and no quantitative electrophysiologic data were provided.^18^

In the present study, peripheral nerve involvement was prospectively investigated in 33 participants who had CPEO (single deletions n = 18 and multiple deletions of nuclear defects n = 15). Based on clinical scores and detailed nerve conduction studies, it could be shown that there was a more severe predominant sensory neuropathy in participants with multiple than with single deletions.

## METHODS

### Participants.

A total of 33 participants with CPEO associated with mtDNA deletions were included (single deletions n = 18 participants and multiple deletions n = 15 participants). The whole period of participants' recruitment was 4 years. Single deletions varied in length between 2 and 6.5 kb (median 5). The common 4.9 kb deletion was identified in 4 patients. The nuclear defects are shown in table 1. The nuclear molecular defect of 1 participant could not be identified (ANT1, Twinkle, POLG1, and POLG2 were investigated). Genetic findings of 3 participants with multiple deletions were published previously.^19,20^ Clinical data of participants with multiple and single deletions are shown in table 2.

**Table 1:**
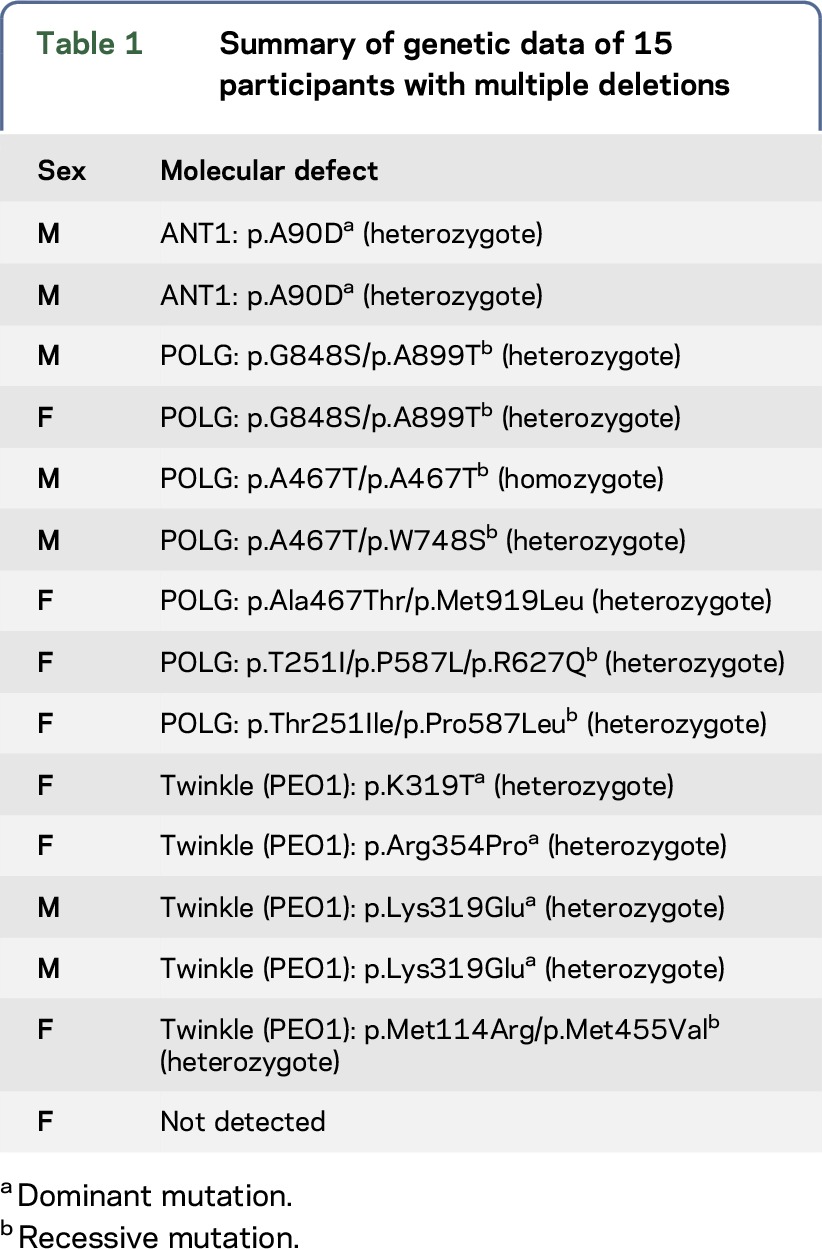
Summary of genetic data of 15 participants with multiple deletions

**Table 2:**
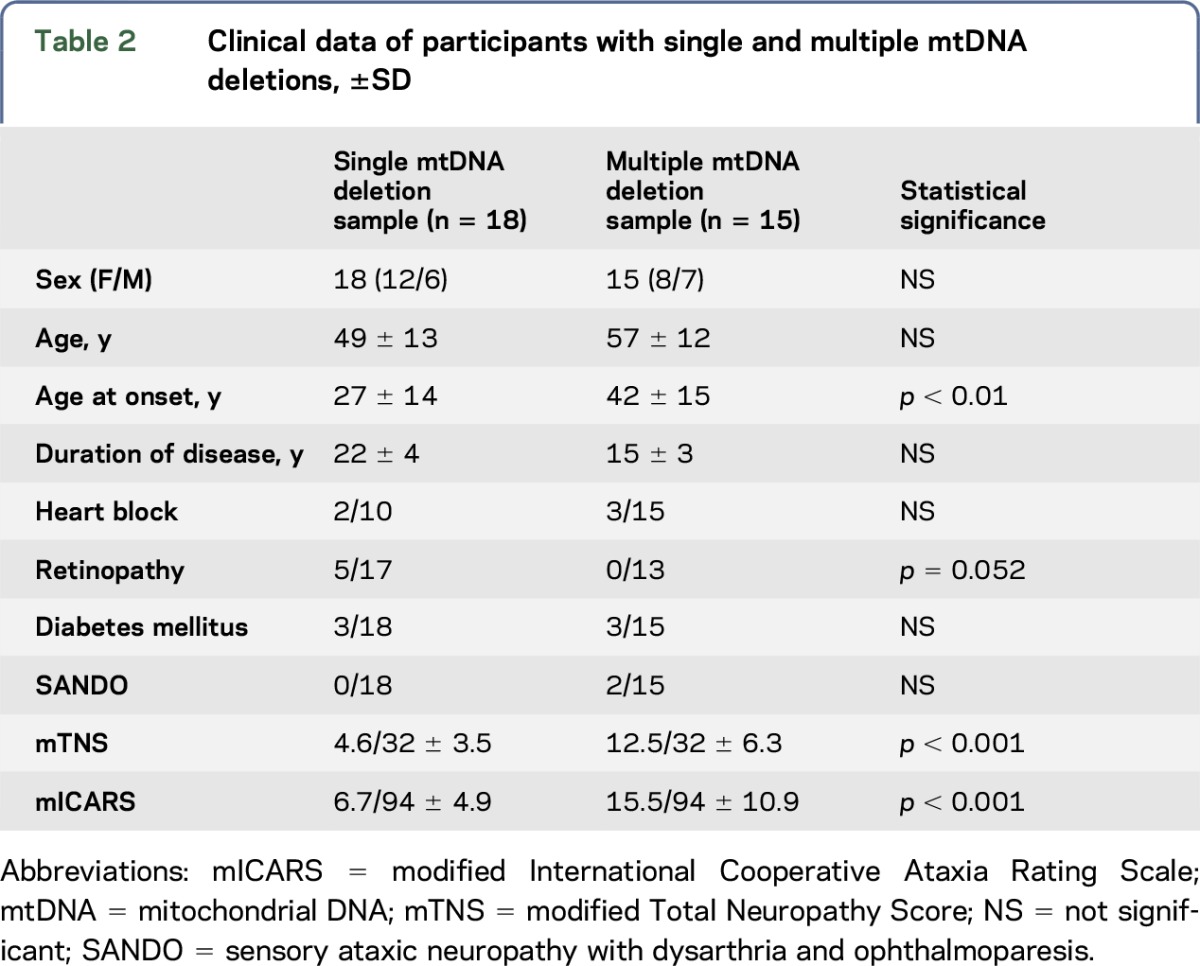
Clinical data of participants with single and multiple mtDNA deletions, ±SD

### Standard protocol approvals and participant consent.

Genetics, electrophysiologic studies, and data analysis were performed in compliance with protocols approved by the Ethical Committee of the Martin Luther University Halle-Wittenberg. Written informed consent was obtained from all participants.

### Procedure.

Multiple and single deletions have been detected by Southern blot analysis and long-range PCR analysis. Genetic studies have been performed according to standard protocols. All 33 participants were clinically characterized using a modified Total Neuropathy Score^21^ (mTNS) (table 3) and a modified International Cooperative Ataxia Rating Scale^22^ (mICARS) (supplemental data at Neurology.org/ng). The modification of mTNS included the following points: the sensibility was examined and rated according to the clinical-neurologic examination; the autonomic symptoms were elicited. mICARS calculation: maximum were 94 points instead of 100 points because examination of eye movement in patients with CPEO had to be omitted. Electrophysiologic studies included nerve conduction studies of sensory nerves (N. suralis and N. sup. radialis), motor nerves (N. tibialis and N. peroneus.), and evoked potentials of the N. tibialis (P40 latency and N35-P40 amplitude values). Nerves without measurable amplitude and therefore without measurable nerve conduction velocity (NCV) in electrophysiologic studies have been omitted from statistical analysis. However, these nerves have been separately indicated in the analysis of the age dependence of the neurographic results. Normal limits for electrophysiologic studies were assessed according to Oh.^23^

**Table 3:**
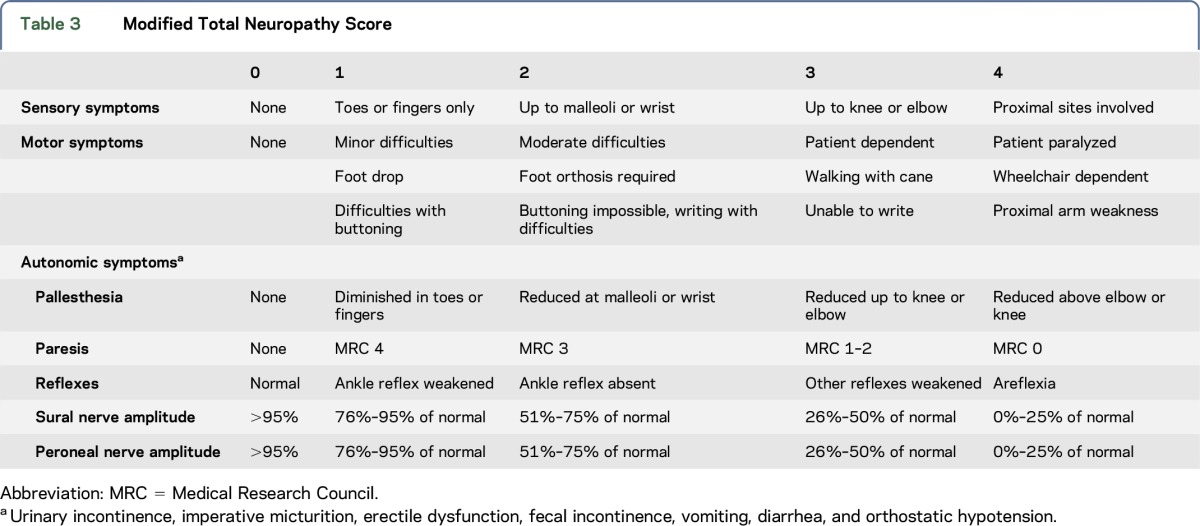
Modified Total Neuropathy Score

## RESULTS

### Clinical scores: mTNS and mICARS.

Participants with single and multiple deletions both had abnormal mTNS and mICARS scores. However, both scores were higher in participants with multiple than those with single deletions (table 2). Two of 15 patients with multiple deletions presented with SANDO phenotype. In these 2 participants, neurography indicated a non–length-dependent neuropathy.

### Neurography.

**Figure 1:**
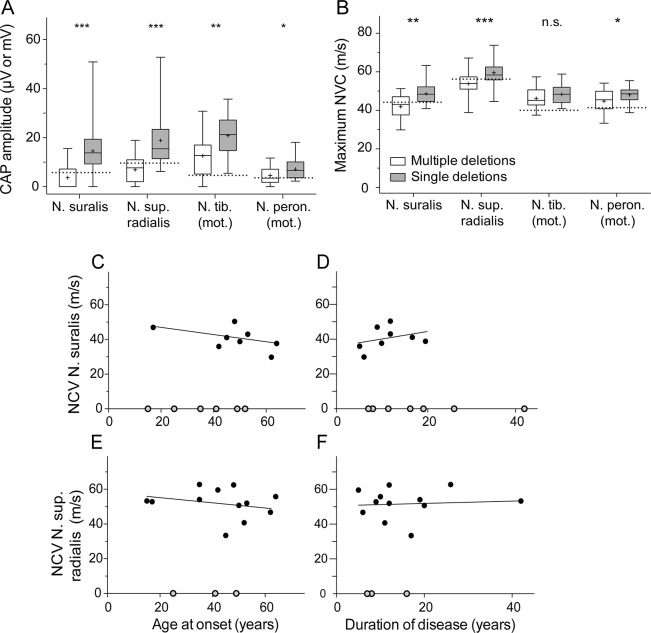
Electrophysiologic studies and age dependence of nerve conduction velocity in multiple deletions (A) Compound action potential (CAP) amplitudes of the N. suralis (single deletions n = 18 and multiple deletions n = 15), N. sup. radialis (single deletions n = 18 and multiple deletions n = 15), N. tibialis (mot.) (single deletions n = 18 and multiple deletions n = 15), and N. peroneus (mot.) (single deletions n = 18 and multiple deletions n = 14). *p < 0.005; **p < 0.0005; ***p < 0.000001 (Mann-Whitney *U* test). Bar shows the range of values. Dotted lines indicate limits of normal according to Oh.^23^ (B) Nerve conduction velocity (NCV) of N. suralis (single deletions n = 16 and multiple deletions n = 8), N. sup. radialis (single deletions n = 18 and multiple deletions n = 12), N. tibialis (mot.) (single deletions n = 18 and multiple deletions n = 14), and N. peroneus (mot.) (single deletions n = 18 and multiple deletions n = 14). *p < 0.05; **p < 0.005; ***p < 0.001 (Mann-Whitney *U* test). n.s. = not statistically significant. Bar shows the range of values. Dotted lines indicate limits of normal according to Oh.^23^ (C) Correlation of age at onset with NCV N. suralis (n = 8): *p* = 0.3, *r* = −0.4. (D) Correlation of duration of disease with NCV N. suralis (n = 8): *p* = 0.3, *r* = 0.5. (E) Correlation of age at onset with NCV N. sup. radialis (n = 12): *p* = 0.1, *r* = −0.5. (F) Correlation of duration of disease and NCV N. sup. radialis (n = 12): *p* = 0.3, *r* = 0.3. Gray dots indicate nerves without measurable amplitude and therefore without measurable NCV.

The differences of CAP and NCV in participants with single and multiple deletions were significant (figure 1, A and B). Sensory CAPs of the sural nerve were absent bilaterally in 7 and unilaterally in further 3/15 participants with multiple mtDNA deletions in contrast to bilateral absence in 2/18 participants with single mtDNA deletions. Early somatosensory evoked potentials of N. tibialis stimulation showed in participants with multiple mtDNA deletions an increase of P40 latency (*p* < 0.005), while the N35-P40 amplitudes (*p* < 0.00005) were reduced, compared with participants with single mtDNA deletions. In participants with multiple deletions, there was no correlation between age at onset or duration of disease with NCVs (figure 1, C–F) or CAP amplitudes (figure e-1). The 2 patients with SANDO were included in the overall cohort for the neuropathy/ataxia scores and all electrophysiologic studies. Reexamination of the data excluding the 2 patients with SANDO did not change the statistical results of the mICARS, mTNS, or electrophysiologic studies.

**Figure 2:**
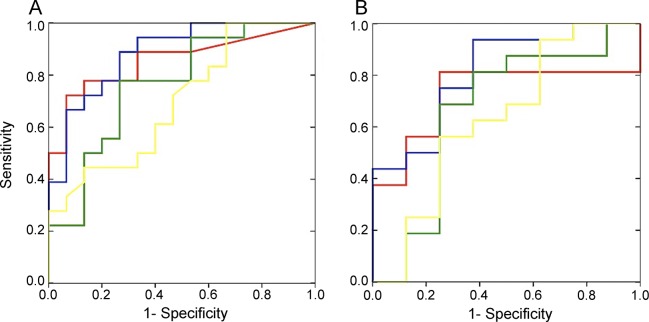
Receiver operating characteristic curve analysis Compound action potential amplitudes (A) and nerve conduction velocities (B): statistical analysis of sensitivity and specificity to detect multiple deletions. N. suralis (red line), N. sup. radialis (blue line), N. tibialis (mot.) (green line), and N. peroneus (mot.) (yellow line).

Statistical analysis of sensitivity and specificity of both sensory nerves (N. suralis and N. sup. radialis) showed the highest AUC (area under the [receiver operating characteristic] curve) levels. However, despite a higher AUC level, the N. suralis showed a better Youden index (figure 2). The decreased CAP amplitudes of the N. suralis had a sensitivity of 93.3% and a specificity of 72.2% (positive predictive value = 73.7% and negative predictive value = 92.9%) to detect multiple deletions.

## DISCUSSION

Peripheral neuropathy is a well-known clinical feature in mitochondrial diseases. In a large phenotype/genotype analysis of 136 patients with CPEO with single mtDNA deletions, polyneuropathy has not been mentioned as a clinical finding.^6^ Another group found peripheral neuropathy in 8% of patients with CPEO with single mtDNA deletions.^5^ However, peripheral neuropathy has also been reported in 32% of patients with multiple mtDNA deletions due to mutations in the gene encoding polymerase gamma as well.^24^ Recently, 1 single deletion patient who had peripheral nerve involvement has been studied.^18^

In the present study, both groups of participants (single and multiple deletions) had higher mTNS and mICARS scores than normal. However, participants with multiple deletions had higher mTNS (*p* < 0.001) and mICARS (*p* = 0.009) values as compared to those with single mtDNA deletions. However, the ICARS is validated for cerebellar disorders only, and high scores can therefore not be considered as proof of sensory ataxia. In fact, patients with CPEO may very well have high scores on the ICARS scale because of myogenic dysarthria or impaired gait due to cerebellar involvement or limb girdle weakness.

Participants with single mtDNA deletions appear to have an earlier age at onset (second decade of life) than patients with multiple mtDNA (age at onset: fourth decade of life). This is consistent with a further study.^25^ Recently, it has been shown that patients with single and multiple mtDNA deletions do not have differences in histopathologic analysis.^26^ Peripheral nerve involvement in multiple deletions did not correlate either with age at onset or duration of disease, indicating that peripheral nerve involvement is a symptom not influenced by age at onset or duration of disease. Thus, the difference in age at onset in multiple and single mtDNA deletions can so far only be stated. The biological explanation remains enigmatic.

Electrophysiologically in both sensory nerves (N. suralis and N. radialis superficialis), CAP amplitudes and NCV were lower and mostly abnormal in participants with multiple deletions. These findings are consistent with those of a previous study.^17^ The 2 motor nerves showed only slight differences. The 2 sensory nerves showed decreased CAP amplitudes and slightly decreased NCV values. This suggests that the nerve involvement is predominantly axonal. Fibers of the radial nerve were affected similarly to those of the sural nerve. Thus, length dependence is questionable in participants with multiple mtDNA deletions. The sensory involvement was supported by early somatosensory evoked potentials, showing that participants with multiple mtDNA deletions had an increase of P40 latency (*p* < 0.005), while the according N35-P40 amplitude values were reduced (*p* < 0.00005).

In a previous study, it has been shown that the number of dorsal root ganglion cells and also posterior horn cells was lower in a patient with pA467T/pX1240Q mutations of the *POLG* gene as compared to that in control subjects.^3^ Anterior horn cells were also relatively low in number in this patient, but the difference to the controls did not reach significance.^3^ Therefore, it can be hypothesized that sensory neuropathy in patients with multiple mtDNA deletions is not only of the axonal type but also might additionally be caused by nerve cell loss.

Recently, it has been shown that peripheral neuropathy in patients with mitochondrial ophthalmoplegia had the highest specificity (91%) for the diagnosis of a nuclear DNA defect.^18^ In the same study, it has been suggested that peripheral neuropathy is a rare finding in patients with single deletions. This is consistent with the present study. Electrophysiologically, both sensory nerves CAP amplitudes and NCV were lower and mostly abnormal in multiple deletions than those in single deletions. However, in the previous study, large fiber peripheral neuropathy was found in only 16/77 patients. Large deletions were seen in 9 of these 16 patients. Of the remaining 5 patients, 1 was a single deletions and 4 were point mutations.^18^ In the present, decreased CAP amplitudes of the sural nerve had a sensitivity of 93.3% and a specificity of 72.2% (positive predictive value = 73.7% and negative predictive value = 92.9%) to detect multiple deletions.

Clinical and electrophysiologic findings of predominant sensory nerve involvement support the notion that sensory neuropathy is not restricted to SANDO syndrome but rather indicates that peripheral sensory nerve involvement is frequent in patients with multiple deletions caused by nuclear defects.

## Supplementary Material

Data Supplement

## GLOSSARY

AUC: area under the curve
CAP: compound action potential
CPEO: chronic progressive external ophthalmoplegia
mICARS: modified International Cooperative Ataxia Rating Scale
mtDNA: mitochondrial DNA
mTNS: modified Total Neuropathy Score
NCV: nerve conduction velocity
SANDO: sensory ataxic neuropathy with dysarthria and ophthalmoparesis

## References

[1] Kaufmann P, Pascual JM, Anziska Y, et al Nerve conduction abnormalities in patients with MELAS and the A3243G mutation. Arch Neurol 2006;63:746–748. 16682545 10.1001/archneur.63.5.746

[2] Garone C, Tadesse S, Hirano M Clinical and genetic spectrum of mitochondrial neurogastrointestinal encephalomyopathy. Brain 2011;134:3326–3332. 21933806 10.1093/brain/awr245 PMC3212717

[3] Lax NZ, Whittaker RG, Hepplewhite PD, et al Sensory neuronopathy in patients harbouring recessive polymerase gamma mutations. Brain 2012;135:62–71. 22189570 10.1093/brain/awr326 PMC3267986

[4] Molnar M, Zanssen S, Buse G, Schroder JM A large-scale deletion of mitochondrial DNA in a case with pure mitochondrial myopathy and neuropathy. Acta Neuropathol 1996;91:654–658. 8781666 10.1007/s004010050480

[5] Laforet P, Lombes A, Eymard B, et al Chronic progressive external ophthalmoplegia with ragged-red fibers: clinical, morphological and genetic investigations in 43 patients. Neuromuscul Disord 1995;5:399–413. 7496174 10.1016/0960-8966(94)00080-s

[6] Yamashita S, Nishino I, Nonaka I, Goto Y Genotype and phenotype analyses in 136 patients with single large-scale mitochondrial DNA deletions. J Hum Genet 2008;53:598–606. 18414780 10.1007/s10038-008-0289-8

[7] Chu CC, Huang CC, Fang W, Chu NS, Pang CY, Wei YH Peripheral neuropathy in mitochondrial encephalomyopathies. Eur Neurol 1997;37:110–115. 9058067 10.1159/000117420

[8] Eymard B, Penicaud A, Leger JM, et al Clinical and electrophysiologic study of the peripheral nerve in 28 cases of mitochondrial disease [in French]. Revue Neurol (Paris) 1991;147:508–512. 1660183

[9] Schubert M, Zierz S, Dengler R Central and peripheral nervous system conduction in mitochondrial myopathy with chronic progressive external ophthalmoplegia. Electroencephalogr Clin Neurophysiol 1994;90:304–312. 7512912 10.1016/0013-4694(94)90149-x

[10] Girlanda P, Toscano A, Nicolosi C, et al Electrophysiological study of neuromuscular system involvement in mitochondrial cytopathy. Clin Neurophysiol 1999;110:1284–1289. 10423194 10.1016/s1388-2457(98)00041-8

[11] Mizusawa H, Ohkoshi N, Watanabe M, Kanazawa I Peripheral neuropathy of mitochondrial myopathies. Revue Neurol (Paris) 1991;147:501–507. 1660182

[12] Yiannikas C, McLeod JG, Pollard JD, Baverstock J Peripheral neuropathy associated with mitochondrial myopathy. Ann Neurol 1986;20:249–257. 3019229 10.1002/ana.410200211

[13] Peyronnard JM, Charron L, Bellavance A, Marchand L Neuropathy and mitochondrial myopathy. Ann Neurol 1980;7:262–268. 6252825 10.1002/ana.410070310

[14] Fadic R, Russell JA, Vedanarayanan VV, Lehar M, Kuncl RW, Johns DR Sensory ataxic neuropathy as the presenting feature of a novel mitochondrial disease. Neurology 1997;49:239–245. 9222196 10.1212/wnl.49.1.239

[15] Santoro L, Manganelli F, Lanzillo R, et al A new POLG1 mutation with PEO and severe axonal and demyelinating sensory-motor neuropathy. J Neurol 2006;253:869–874. 16715201 10.1007/s00415-006-0082-6

[16] Bardosi A, Friede RL, Ropte S, Goebel HH A morphometric study on sural nerves in metachromatic leucodystrophy. Brain 1987;110:683–694. 3580829 10.1093/brain/110.3.683

[17] Hanisch F, Kornhuber M, Alston CL, Taylor RW, Deschauer M, Zierz S SANDO syndrome in a cohort of 107 patients with CPEO and mitochondrial DNA deletions. J Neurol Neurosurg Psychiatry 2015;86:630–634. 25143630 10.1136/jnnp-2013-306748

[18] Horga A, Pitceathly RD, Blake JC, et al Peripheral neuropathy predicts nuclear gene defect in patients with mitochondrial ophthalmoplegia. Brain 2014;137:3200–3212. 25281868 10.1093/brain/awu279 PMC4240292

[19] Deschauer M, Hudson G, Muller T, Taylor RW, Chinnery PF, Zierz S A novel ANT1 gene mutation with probable germline mosaicism in autosomal dominant progressive external ophthalmoplegia. Neuromuscul Disord 2005;15:311–315. 15792871 10.1016/j.nmd.2004.12.004

[20] Hudson G, Deschauer M, Busse K, Zierz S, Chinnery PF Sensory ataxic neuropathy due to a novel C10Orf2 mutation with probable germline mosaicism. Neurology 2005;64:371–373. 15668446 10.1212/01.WNL.0000149767.51152.83

[21] Cornblath DR, Chaudhry V, Carter K, et al Total neuropathy score: validation and reliability study. Neurology 1999;53:1660–1664. 10563609 10.1212/wnl.53.8.1660

[22] Trouillas P, Takayanagi T, Hallett M, et al International Cooperative Ataxia Rating Scale for pharmacological assessment of the cerebellar syndrome. The Ataxia Neuropharmacology Committee of the World Federation of Neurology. J Neurol Sci 1997;145:205–211. 9094050 10.1016/s0022-510x(96)00231-6

[23] Oh SJ Clinical Electromyography: Nerve Conduction Studies, 3rd ed Philadelphia: Lippincott Williams & Wilkins; 2003.

[24] Horvath R, Hudson G, Ferrari G, et al Phenotypic spectrum associated with mutations of the mitochondrial polymerase gamma gene. Brain 2006;129:1674–1684. 16621917 10.1093/brain/awl088

[25] Kawai H, Akaike M, Yokoi K, et al Mitochondrial encephalomyopathy with autosomal dominant inheritance: a clinical and genetic entity of mitochondrial diseases. Muscle Nerve 1995;18:753–760. 7783765 10.1002/mus.880180712

[26] Zierz CM, Joshi PR, Zierz S Frequencies of myohistological mitochondrial changes in patients with mitochondrial DNA deletions and the common m.3243A>G point mutation. Neuropathology 2015;35:130–136. 25378026 10.1111/neup.12173

