# Supplementary material for: Peripheral neuropathy in patients with CPEO associated with single and multiple mtDNA deletions
Source: Neurol Genet. 2016 Oct 19;2(6):e113. doi: 10.1212/NXG.0000000000000113 (PMC5089902; doi:10.1212/NXG.0000000000000113)
Supplement: Data Supplement [file supp_2_6_e113__index.html]

Data Supplement 

# Peripheral neuropathy in patients with CPEO associated with single and multiple mtDNA deletions

## Data Supplement

**Files in this Data Supplement:**

- Figure e-1
- mICARS
