# Supplementary material for: Peripheral neuropathy in patients with CPEO associated with single and multiple mtDNA deletions
Source: Neurol Genet. 2016 Oct 19;2(6):e113. doi: 10.1212/NXG.0000000000000113 (PMC5089902; doi:10.1212/NXG.0000000000000113)
Supplement: Data Supplement [file supp_2.6.e113_Figure_e-1.docx]

**Suppl. Fig.:** Age dependence of CAP amplitudes in multiple deletions: **A:** Correlation of age at onset with amplitude N. suralis (n=8): ns; **B:** Correlation of duration of disease with amplitude N. suralis (n=8): ns; **C:** Correlation of age at onset with amplitude N. sup. radialis (n=12): ns; **D:** Correlation of duration of disease and amplitude N. sup. radialis (n=12): ns*. Grey dots: Nerves without measurable amplitude. ns= not significant.*
