# Supplementary material for: Peripheral neuropathy in patients with CPEO associated with single and multiple mtDNA deletions
Source: Neurol Genet. 2016 Oct 19;2(6):e113. doi: 10.1212/NXG.0000000000000113 (PMC5089902; doi:10.1212/NXG.0000000000000113)
Supplement: Data Supplement [file supp_2.6.e113_mICARS.docx]

**Modified International Cooperative Ataxia Rating Scale (mICARS)**

**I. Posture and Gait disturbance**

*1. Walking capacities*

0 normal

1 almost normal naturally, but unable to walk with feet in tandem position

2 Walking without support, but clearly abnormal and irregular

3 Walking without support but with considerable staggering, diffculties in half turn

4 Walking with autonomous support no longer possible, the patient uses episodic support of the wall

5 Walking only possible with one stick

6 Walking only possible with two special sticks or with a stroller

7 Walking only with accompanying person

8 walking impossible even with accompanying person (wheelchair)

*2. Gait Speed*

0 normal

1 slightly reduced

2 markedly reduced

3 extremely slow

4 walking with autonomous support no longer possible

*3. Standing Capacities with eyes open*

0 normal, able to stand on one foot more than 10 sec

1 able to stand with feet together, but no longer able to stand on one foot more than 10 sec.

2 able to stand with feet together, but no longer able to stand in tandem position

3 no longer able to stand with feet together, but able to stand in natural position without support, with no or

moderate sway

4 standing in natural position without support, with considerable sway and considerable corrections

5 unable to stand in natural position without strong support of the arms

6 unable to stand at all, even with string support of the arms

*4. Spread of feet in natural position without support, eyes open*

0 normal (<10cm)

1 >10cm

2 25-35cm

3 >35cm

4 standing in natural position impossible

*5. Body sway with feet together, eyes open*

0 normal

1 slightly oscillations

2 moderate oscillations (<10cm at the level of head)

3 severe oscillations (>10cm at the level of head), threatening the upright position

4 immediate falling

*6. Body sway with feet together, eyes closed*

0 normal

1 slightly oscillations

2 moderate oscillations (<10cm at the level of head)

3 severe oscillations (>10cm at the level of head), threatening the upright position

4 immediate falling

*7. Quality of sitting position*

0 normal

1 with slight oscillations of the trunk

2 with moderate oscillations of the trunk and legs

3 with severe dysequilibrium

4 impossible

**II. Kinetic Functions**

*8. Knee-Tibia Test*

0 normal

1 lowering of heel in continuous axis, but the movement is decomposed in several phases, without real jerks, or

abnormally slow

2 lowering jerkily in the axis

3 lowering jerkily with lateral movements

4 lowering jerkily with extremely strong lateral movements or test impossible

*9. Action tremor in Heel to Knee Test*

0 normal

1 Tremor stopping immediately when the heel reaches the knee

2 Tremor stopping in less than 10 seconds after reaching the knee

3 Tremor continuing for more than 10 seconds after reaching the knee

4 uninterrupted tremor or test impossible

*10. Finger to nose Test*: *decomposition and dysmetria*

0 normal

1 Oscillating movement without decomposition of the movement

2 Segmented movement in more than 2 phases and/or moderate dysmetria in reaching nose 3 segmented movement in more than 2 phases and/or considerable dysmetria in reaching

nose

4 Dysmetria preventing the patient from reaching the nose

*11. Finger to nose Test: intention tremor*

0 normal

1 simple swerve of the movement

2 moderate tremor with estimated amplitude <10cm

3 Tremor with estimated amplitude between 10cm und 40cm

4 severe tremor with estimated amplitude >40cm

*12. Finger-Finger-Test: action tremor and/or instability*

0 normal

1 mild instability

2 moderate oscillations of finger with estimated amplitude <10cm

3 considerable oscillations of finger with estimated amplitude between 10 and 40cm

4 Jerky movement >40cm of amplitude

*13. Pronation-Supination: altering movements*

0 normal

1 slightly irregular and slowed

2 clearly irregular and slowed, but without sway of the elbow

3 extremely irregular and slowed movement, with sway of the elbow

4 movement completely disorganized or impossible

*14. Drawing the Archimedes spiral*

0 normal

1 impairment and decomposition, the line quitting the pattern slightly, but without hypermetric swerve

2 line completely out of the pattern without recrossing and/or hypermetric swerves

3 major disturbance due to hypermetria and decomposition

4 drawing completely disorganised or impossible

**III. Speech**

*15. Dysarthria: fluency of speech*

0 normal

1 mild modification of fluency

2 moderate modification of fluency

3 considerably slow and dysarthric speech

4 no speech

*16. Dysarthria: clarity of speech*

0 normal

1 suggestion of slurring

2 definite slurring, most words understandable

3 severe slurring, speech not understandable

4 no speech
